# The importance of mean time in therapeutic range for complication rates in warfarin therapy of patients with atrial fibrillation: A systematic review and meta-regression analysis

**DOI:** 10.1371/journal.pone.0188482

**Published:** 2017-11-20

**Authors:** Anne Sig Vestergaard, Flemming Skjøth, Torben Bjerregaard Larsen, Lars Holger Ehlers

**Affiliations:** 1 Danish Center for Healthcare Improvements, Department of Business and Management, Faculty of Social Sciences, Aalborg University, Aalborg, Denmark; 2 Unit of Clinical Biostatistics, Aalborg University Hospital, Aalborg, Denmark; 3 Aalborg Thrombosis Research Unit, Department of Clinical Medicine, Faculty of Health, Aalborg University, Aalborg Denmark; 4 Department of Cardiology, Aalborg University Hospital, Aalborg, Denmark; Inselspital Universitatsspital Bern, SWITZERLAND

## Abstract

**Background:**

*\*Anticoagulation is used for stroke prophylaxis in non-valvular atrial fibrillation, amongst other by use of the vitamin K antagonist, warfarin. Quality in warfarin therapy is often summarized by the time patients spend within the therapeutic range (percent time in therapeutic range, TTR). The correlation between TTR and the occurrence of complications during warfarin therapy has been established, but the influence of patient characteristics in that respect remains undetermined. The objective of the present papers was to examine the association between mean TTR and complication rates with adjustment for differences in relevant patient cohort characteristics.

**Methods:**

A systematic literature search was conducted in MEDLINE and Embase (2005–2015) to identify eligible studies reporting on use of warfarin therapy by patients with non-valvular atrial fibrillation and the occurrence of hemorrhage and thromboembolism. Both randomized controlled trials and observational cohort studies were included. The association between the reported mean TTR and major bleeding and stroke/systemic embolism was analyzed by random-effects meta-regression with and without adjustment for relevant clinical cohort characteristics. In the adjusted meta-regressions, the impact of mean TTR on the occurrence of hemorrhage was adjusted for the mean age and the proportion of populations with prior stroke or transient ischemic attack. In the adjusted analyses on thromboembolism, the proportion of females was, furthermore, included.

**Results:**

Of 2169 papers, 35 papers met pre-specified inclusion criteria, holding relevant information on 31 patient cohorts. In univariable meta-regression, increasing mean TTR was significantly associated with a decreased rate of both major bleeding and stroke/systemic embolism. However, after adjustment mean TTR was no longer significantly associated with stroke/systemic embolism. The proportion of residual variance composed by between-study heterogeneity was substantial for all analyses.

**Conclusions:**

Although higher mean TTR in warfarin therapy was associated with lower complication rates in atrial fibrillation, the strength of the association was decreased when adjusting for differences in relevant clinical characteristics of the patient cohorts. This study suggests that mainly the safety of warfarin therapy increases with higher mean TTR, whereas effectiveness appears not to be substantially improved.

Due to the limitations immanent in the meta-regression methods, the results of the present study should be interpreted with caution. Further research on the association between the quality of warfarin therapy and risk of complications is warranted with adjustment for clinically relevant characteristics.

## Introduction

Non-valvular atrial fibrillation (AF) is the most common cardiac arrhythmia with a prevalence as high as 1.5–2.0% in the general population[1]. AF increases the risk of stroke substantially, why effective stroke prophylaxis is vital[2]. Guidelines recommend continuous anticoagulation for the majority of AF patients, either by non-vitamin K antagonist oral anticoagulants (NOACs) or vitamin K antagonists, such as warfarin[2,3]. Particularly warfarin therapy may be challenging due to a narrow therapeutic range; outside it, patients are exposed to an increased risk of either thromboembolism or hemorrhage. A generally accepted quality measure in warfarin therapy is the time patients spend within the therapeutic range (percent time in therapeutic range; TTR)[3,4]. An inverse correlation between TTR and hemorrhagic and thromboembolic complications has been established and it has been suggested that the benefits of warfarin therapy may be outweighed if the quality of warfarin therapy is too poor[5–8]. In continuation hereof, it may be beneficial to increase TTR, if possible, as it would decrease the risk of therapy-related complications.

In addition, the risk of hemorrhage and thromboembolism also depends on patient characteristics, such as sex, age, and prior bleeding and thromboembolism[2]. The presence of these risk factors in a patient population may complicate the assumption of a ‘simple’ association between the mean TTR of patient cohorts and complications as the relationship may be confounded by the clinical characteristics of the patient cohorts. The purpose of the present study was to evaluate the association between mean TTR of AF patient populations on warfarin therapy and the occurrence of patient-relevant outcomes through the execution of a systematic review of randomized controlled trials (RCTs) and observational cohort studies and subsequent meta-regression. The analysis is conducted as a random-effects meta-regression, estimating the effect of increasing mean TTR on complication rates with and without adjustment for clinically relevant variables.

## Materials and methods

### Search strategy and eligibility criteria

A systematic review was performed of Embase and MEDLINE for papers published in the period from January 2005 through 2015. The literature search was performed on 2 February 2016. The aim of the literature search and systematic review was to identify studies reporting on use of warfarin therapy in patients with non-valvular AF and the occurrence of thromboembolism and hemorrhage. The search strategy included use of thesaurus terms and explosions of the same words. Additional studies were identified through prior knowledge of the literature and snowballing on the reference lists of already acquired papers[5,9]. Eligible studies fulfilled the following inclusion criteria; full-text papers, in Danish or English, on RCTs or observational cohort studies; both retrospective and prospective studies were included. When multiple papers reported on the same patient cohort, the study with the greatest level of detail on parameters of interest was included; for instance, a study reporting on complication rates of multiple TTR strata would be chosen over another study on the entire cohort. Studies should report on anticoagulation with dose-adjusted warfarin (target therapeutic range; international normalized ratio (INR) of 2–3) as the predominantly used vitamin K antagonist for adult patients (age>18 years) with non-valvular AF as main indication. Studies on populations, of which a minor proportion of patients might have a concomitant indication for anticoagulant therapy, such as deep venous thrombosis or other, were included. Studies on patients with valvular AF were excluded as were studies in which complication rates were reported aggregately for mixed populations, for instance for patients with valvular and non-valvular AF combined. Quality of therapy should be reported by information on the study populations’ TTR. Studies reporting on specific subpopulations were excluded if generalizability of the results to the broader AF population could not be expected, as were studies reporting on anticoagulation in relation to surgery or other therapies. Studies should include information on the occurrence of thromboembolism and/or hemorrhage. Only studies with more than 50 persons in each treatment group were included, and the mean follow-up time of patients should be more than 3 months. The systematic review is reported in accordance with the PRISMA (Preferred Reporting Items for Systematic reviews and Meta-Analyses) guidelines[10] (S1 Table. PRISMA 2009 Checklist). The full electronic search strategy is shown in S2 Table. The rationale for the applied eligibility criteria is given in S3 Table.

### Study selection and data extraction

The assessment of the eligibility of studies and data extraction was performed by the first author (ASV) of the present study. Titles and abstracts of the identified papers were reviewed and papers that did not meet the above-mentioned inclusion criteria were excluded. Papers deemed potentially relevant by evaluation of the title and abstract were retrieved in full text for further assessment. Papers were subsequently excluded if they did not meet the stated inclusion criteria based on the full-text assessment. The following information was retrieved from studies; first author, publication year, study setting; RCT versus ‘real-life’; i.e. anticoagulation clinic or community practice (RCTs were defined as studies in which a randomization of patients into groups had been performed, anticoagulation clinic were defined as studies undertaken at solely anticoagulation clinics, but without randomization; community practice included all other studies), whether the study was reported as being prospective or retrospective, geographical location of study (North America, Europe including the United Kingdom, multinational, or ‘other’), total number of patient-years, number of patients treated, method for estimation of TTR, mean age and TTR of the patient population, characteristics of the study population in terms of proportion that was female, had prior stroke or transient ischemic attack (TIA), and that had a CHADS_2_ (Congestive heart failure, hypertension age>75, diabetes mellitus, prior stroke, TIA, or systemic embolism[doubled]) score of 0–1, 2, and 3+, respectively. When information on clinical characteristics was provided for an entire study population, but event rates were stated for strata, the characteristics of the entire study population were assumed for the strata.

For studies, in which the safety and effectiveness of warfarin were compared to that of another oral anticoagulant agent, information was retrieved only for the group treated with warfarin. Information on events was retrieved as rates, per 100 patient-years. All information was retrieved as mean values or proportions, when possible. When median values were provided, these were assumed to be similar to the mean. When intervals for variables were given, such as TTR and age, mean values for these were applied as point estimates. If relevant data were not readily available in papers, they were estimated based on the provided data, for instance, total number of patient-years was estimated by multiplying the number of patients with the mean/median follow-up time per patient (Table 1). When necessary, event rates were estimated based on total number of events and total follow-up time to allow comparability between studies. Primary outcomes included major bleeding (MB) and stroke/systemic embolism (SSE). In general, the definition of MB included intracranial and extracranial hemorrhage requiring hospitalization, blood transfusion, surgical treatment, or when it occurred at a critical anatomical site. Supplementary analyses were performed for hemorrhagic stroke (HS) and ischemic stroke (IS). For the present study, events were included as defined in papers.

**Table 1:** Study characteristics.

Study	Study design/setting	Location	Patient-years	N	Mean TTR, %	Mean age	Proportion female, %	Proportion prior stroke /TIA, %	CHADS_2_ 0–1, %	CHADS_2_ 2, %	CHADS_2_ 3+, %	MB	SSE	HS	IS
Abdelhafiz 2008, age<75[23]	PD/AC	European	321	203	58	64	37.9	18.7	-	-	-	1.56	-	0.00	-
Abdelhafiz 2008, age≥75[23]	PD/AC	European	315	199	58	80	50.8	30.2	-	-	-	1.90	-	0.00	-
Büller 2008[24]	PD/RCT	Multinational	2271	2293	63	70	34.5	25.1	39.6	32.2	28.1	1.38	1.28	0.24	0.95
Burton 2006[25]	RD/community	European	953	309	68	77	48.9	21.0	-	-	-	2.60	-	-	1.64
Connolly 2006[21]†	PD/RCT	Multinational	4315*	3371	64	70	34.4	15.1	-	-	-	2.21‡	-	0.36	1.00‡
Connolly 2008, TTR<53.8%[6]	PD/RCT	Multinational	863*	674	44	70	35.9	15.1	-	-	-	2.92	1.95	-	1.95
Connolly 2008, TTR 53.8%-65.0%[6]	PD/RCT	Multinational	1185*	926	60	70	35.9	15.3	-	-	-	2.36	1.23	-	1.23
Connolly 2008, TTR 65.1%-73.3%[6]	PD/RCT	Multinational	1285*	1004	69	71	33.0	15.0	-	-	-	1.95	1.40	-	1.25
Connolly 2008, TTR>73.3[6]	PD/RCT	Multinational	982	767	78	71	33.0	15.0	-	-	-	1.78	1.47	-	1.36
Connolly 2013[26]	PD/RCT	Multinational	51*	127	63	73	29.9	-	29.1	33.1	37.8	5	0	0	0
Currie 2005, (stable)[27]	RD/community	UK	2274*	784	75	74	46.2	-	-	-	-	0.4	-	-	-
Currie 2005, (unstable)[27]	RD/community	UK	2114*	729	56	78	47.5	-	-	-	-	1.2	-	-	-
Giugliano 2013[28]	PD/RCT	Multinational	19687*	7036	65	72§	37.5	28.3	-	-	-	3.43	1.80	0.47	1.25
Hylek 2007, total[29]	PD/AC	N. American	360	472	58	77	47.0	5.3	34.5	38.3	27.1	7.22‡	-	1.67	-
Hylek 2007, age<80[29]	PD/AC	N. American	253	319	58|	73	43.3	3.5	46.4	34.8	18.8	4.75	-	-	-
Hylek 2007, age≥80[29]	PD/AC	N. American	107	153	58|	84	54.9	9.2	9.8	45.8	44.4	13.08	-	-	-
Jacobs 2009[30]	RD/community	N. American	90	90	49	82	77.8	-	-	-	-	6	-	-	2
Lip 2015[31]	PD/RCT	Multinational	167	324	48	66	34.9	-	35.8	39.2	25.0	4.78	-	0.60	-
Mant 2007[32]	PD/RCT	UK	1318*	488	67	82	45.3	13.1	-	-	-	1.90	0.83	0.46	0.76
Menzin 2005, site A[33]	RD/AC	N. American	168*	200	60	74	45.5	14.0	-	-	-	4.16	-	-	0.00
Menzin 2005, site B[33]	RD/AC	N. American	173*	200	61	72	39.5	18.5	-	-	-	2.31	-	-	0.58
Menzin 2005, site C[33]	RD/AC	N. American	183*	200	65	71	46.0	16.5	-	-	-	3.82	-	-	2.18
Nichol 2008, community[34]	RD/community	N. American	1163*	756	42	-	45.9	-	-	-	-	6.27	-	-	3.73
Nichol 2008, AC[34]	RD/AC	N. American	920*	351	68	-	42.5	-	-	-	-	2.25	-	-	1.93
Njaastad 2006[35]	RD/AC	European	475	421	72	75§	43.6	-	-	-	-	0.84	2.10	-	2.10
Pastori 2015[36]	PD/AC	European	1755	627	67	73	40.2	14.2	-	-	-	1.82	-	-	-
Pengo 2010[37]	PD/RCT	Multinational	697*	132	65	79	59.1	0.0	-	-	-	3.01	2.01	-	2.01
Patel 2011[19]†	PD/RCT	Multinational	13807*	7133	55	73§	39.7	54.6	0	13.1	86.9	3.4	-	0.44	1.42
Piccini 2014, TTR 0–50.6%[20]	PD/RCT	Multinational	3269*	1689	25¶	70§	43.9	58.2	0	10.6	89.4	-	2.0	-	-
Piccini 2014, TTR 50.7–58.5%[20]	PD/RCT	Multinational	3498*	1807	55¶	72§	40.7	53.1	0	10.7	89.3	-	1.6	-	-
Piccini 2014, TTR 58.6–65.7%[20]	PD/RCT	Multinational	3403*	1758	62¶	74§	39.1	51.4	0	12.8	87.2	-	1.6	-	-
Piccini 2014, TTR 65.8–100%[20]	PD/RCT	Multinational	3535*	1826	83¶	75§	34.9	47.2	0	18.3	81.7	-	1.3	-	-
Poli 2007, age 75–79[38]	PD/AC	European	433	-	69|	77¶	40.3|	38.3|	14.5|	28.6|	56.9|	1.39	-	0.69	-
Poli 2007, age 80–84[38]	PD/AC	European	271	-	69|	82¶	40.3|	38.3|	14.5|	28.6|	56.9|	2.58	-	2.21	-
Poli 2007, age 85–96[38]	PD/AC	European	110	-	69|	91¶	40.3|	38.3|	14.5|	28.6|	56.9|	3.64	-	1.82	-
Poli 2009, total[39]	PD/AC	European	2567	783	71§	75§	35.2	29.2	28.6	29.2	42.1	1.44‡	-	0.7‡	0.9
Poli 2009, age<80[39]	PD/AC	European	1738	456	71§|	73§	31.4	24.6	40.8	27.2	32.0	0.92	-	0.58	-
Poli 2009, age≥80[39]	PD/AC	European	829	327	71§|	83§	40.7	35.8	11.6	32.1	56.3	2.53	-	1.21	-
Poli 2009[40]	PD/AC	European	1854	578	68	75§	36.7	34.0	-	-	-	-	-	-	0.97
Poli 2011[41]	PD/AC	European	10019	3302	68§	74§	44.7	-	-	-	-	1.24	-	0.27	0.41
Reddy 2014[42]	PD/RCT	Multinational	903*	244	70	73	29.9	20.1	27.0	36.1	36.9	3.1	1.1	1.1	1.1
Rose 2008, total[43]	PD/community	N. American	2892	3104	67	74	41.9	10.8	50.6	29.4	20.0	1.90‡	1.00	-	-
Rose 2008, TTR<60%[43]	PD/community	N. American	881	1141	30¶	74	46.5	12.0	47.9	30.6	21.5	3.06	-	-	-
Rose 2008, TTR≥60%, TTR≤75%[43]	PD/community	N. American	917	1009	68¶	75	42.6	9.1	50.5	29.7	19.8	1.53	-	-	-
Rose 2008, TTR>75%[43]	PD/community	N. American	1093	1246	88¶	74	37.9	10.4	52.7	28.9	18.4	1.28	-	-	-
Sanden 2015, TTR 60%-70%[44]	RD/community	European	410	-	65¶	72|	38.5|	25.0|	-	-	-	2.68	3.65	0.00	-
Sanden 2015, TTR 70%-75%[44]	RD/community	European	36770	-	73¶	72|	38.5|	25.0|	-	-	-	2.21	1.49	0.37	-
Sanden 2015, TTR 75%-80%[44]	RD/community	European	81712	-	78¶	72|	38.5|	25.0|	-	-	-	2.17	147	0.38	-
Sanden 2015, TTR 80%-100%[44]	RD/community	European	23727	-	90¶	72|	38.5|	25.0|	-	-	-	2.15	1.56	0.42	-
Singer 2009[45]	RD/PD/community	N. American	32130/33415#	7206	65	73§	40.8	12.3	-	-	-	-	1.27	0.29	-
Sullivan 2013, women[46]	PD/RCT	Multinational	5547*	1594	60	71	100	13.3	-	-	-	-	-	-	5
Sullivan 2012, men[46]	PD/RCT	Multinational	8582*	2466	63	68	0	13.4	-	-	-	-	-	-	3
Tincani 2009[47]	PD/AC	European	90	90	66	92§	76.7	36.7	-	-	-	5.56	1.11	1.11	1.11
Turk 2015[48]	PD/AC	Turkish	789*	403	40	70	56.3	12.2	-	-	-	2.91	-	-	-
Connolly 2009[16]†	PD/RCT	Multinational	12044*	6022	64	72	36.7	19.8	30.9	37.0	32.1	3.36‡	1.69‡	0.38	1.20
Wallentin 2010, TTR<57.1%[15]	PD/RCT	Multinational	3008*	1504	51§	70	40.1	15.4	28.0	36.8	35.3	3.59	1.63	-	-
Wallentin 2010, TTR 57.1%-65.5%[15]	PD/RCT	Multinational	3028*	1514	63§	71	35.5	12.9	31.9	34.9	33.1	4.13	1.63	-	-
Wallentin 2010, TTR 65.5%-72.6%[15]	PD/RCT	Multinational	2974*	1487	70§	72	34.6	11.8	32.1	35.0	32.9	3.40	1.11	-	-
Wallentin 2010, TTR>72.6%	PD/RCT	Multinational	3018*	1509	79§	73	35.6	10.3	35.4	36.0	28.6	3.11	0.97	-	-
Granger 2011[18]†	PD/RCT	Multinational	16346*	9081	62	70§	35.0	19.7	34.0	35.8	30.2	3.09‡	1.60‡	0.47	1.05
Wallentin 2013, TTR 24.3%-60.5%[17]	PD/RCT	Multinational	3922*	2179	51§	68§	39.7	13.8	31.4	35.9	32.7	2.89	2.36	-	-
Wallentin 2013, TTR 60.6%-66.3%[17]	PD/RCT	Multinational	4003*	2224	63§	69§	38.0	12.6	33.6	35.2	31.3	3.07	1.72	-	-
Wallentin 2013, TTR 66.4%-71.1%[17]	PD/RCT	Multinational	4032*	2240	69§	71§	34.0	11.8	33.5	36.1	30.4	3.06	1.35	-	-
Wallentin 2013, TTR 71.2%-83.2%[17]	PD/RCT	Multinational	4007*	2226	77§	72§	29.5	8.6	37.4	36.1	26.5	3.31	1.04	-	-
White 2007, TTR<60%[49]	PD/RCT	Multinational	1646*	1190	48§	71	31.3	21.8	-	-	-	-	2.10	0.20	1.84
White 2007, TTR≥60%, TTR≤75%[49]	PD/RCT	Multinational	1670*	1207	68§	71	31.3	21.5	-	-	-	-	1.34	0.28	1.06
White 2007, TTR>75%[49]	PD/RCT	Multinational	1646*	1190	83§	71	28.2	18.1	-	-	-	-	1.07	0.06	1.02

AC: Anticoagulation clinic, CHADS_2:_ See body text, HS: Hemorrhagic stroke, IS: Ischemic stroke, MB: Major bleeding, PD: Prospective design, RCT: Randomized controlled trial, RD: Retrospective design, SSE: Stroke/systemic embolism, TIA: transient ischemic attack, TTR: Time in therapeutic range.

*Estimated from mean/median number of follow-years per patient

†More papers based on the same patient cohort, but reporting on different outcomes

‡Data excluded from analysis due to disaggregate data available from strata in sub-studies

§Median

|Data point from whole sample applied to stratum

¶Point estimate

#Patient-years for HS

### Statistical analysis

Transformation was applied for the outcome rates, as they were non-Gaussian distributed. An underlying binomial distribution of the rate was assumed as no event rates superseded 1. A modified double arcsine transformation was chosen over the logit transformation to avoid otherwise possible overdispersion caused by the rarity of events and to stabilize variances by

$$\theta =2*\sin^{-1}\left(\sqrt{rate}\right)$$

in which *θ* is the double arcsine transformed rate[11]. Standard errors of the transformed rates were estimated as

$${SE}_{\theta }=\sqrt{\frac{1}{\left(pt\;year+0.5\right)}}.$$

All analyses were performed on the double arcsine transformed rates. For illustrations, results were back-transformed by

$$rate={\left(\sin\frac{\theta }{2}\right)}^{2}$$

and reported on the original scale.[11]

The risk of small-study effects and publication bias was assessed by inspection of funnel plots of the effect size of studies against their estimated standard error within tertiles of mean TTR of studies and Egger’s regression statistics for the same[12]. Between-study heterogeneity was evaluated using the *I*^*2*^ statistics (including the Cochrane *Q* test *p*-value), for which an *I*^*2*^>75% was considered to indicate a large degree of statistical heterogeneity[13].

The correlation between the reported mean TTR and the double arcsine transformed outcome rates was evaluated by univariable, linear, random-effects meta-regressions with mean TTR as the only predictor variable. In the random-effects meta-regression, the weight of studies, used to estimate the pooled effect size, is determined by within-study variance plus between-study variance, equalizing the weights of larger and smaller studies[11,13]. The proportion of the total variance explained by between-study heterogeneity was evaluated by the *I*^*2*^_*res*_[13].

In addition, multivariable meta-regressions were performed to adjust for potential effect modification or confounding with inclusion of a priori chosen relevant clinical covariates that differ in real life patient cohorts [2,14]. In the meta-regressions on MB and HS, the impact of mean TTR was adjusted for mean age and the proportion of populations with prior stroke or TIA. In analyses on SSE and IS, the proportion of the study population that was female was, furthermore, included. All covariates were assumed to have a continuous, linear effect. Studies with missing information on the included covariates were excluded from the adjusted analyses. As a result, univariable meta-regressions with mean TTR as single predictor were also performed for the set of studies used in the adjusted analyses. Using the same regression basis, this allowed for comparison of residual between-study heterogeneity in the uni- and multivariable meta-regressions. Permutation tests were performed for all multivariable meta-regressions to adjust for multiplicity[14].

For supplementary analysis, the effect of mean TTR was adjusted for the impact of methodological variables by inclusion of covariates on publication year (grouped into years; 2005–2007, 2008–2011, 2012–2015), setting (RCT, anticoagulation clinic, or community practice), location (North America, European/UK, multinational, or other) and whether the study was prospective, retrospective, or mixed.

2-tailed significance tests were used. Statistical significance was assumed for *p*-values<0.05, except for Egger’s regression statistics for which a 0.1 threshold for significance was applied[12]. All statistical analyses were performed using Stata/MP 13 (StataCorp LP, College Station, TX). The meta-regressions were conducted using the Stata command ‘metareg’ with default settings. The calculated standard errors of the transformed rates of individual studies were employed as within-study standard error (‘wsse’).

## Results

**Fig 1:**
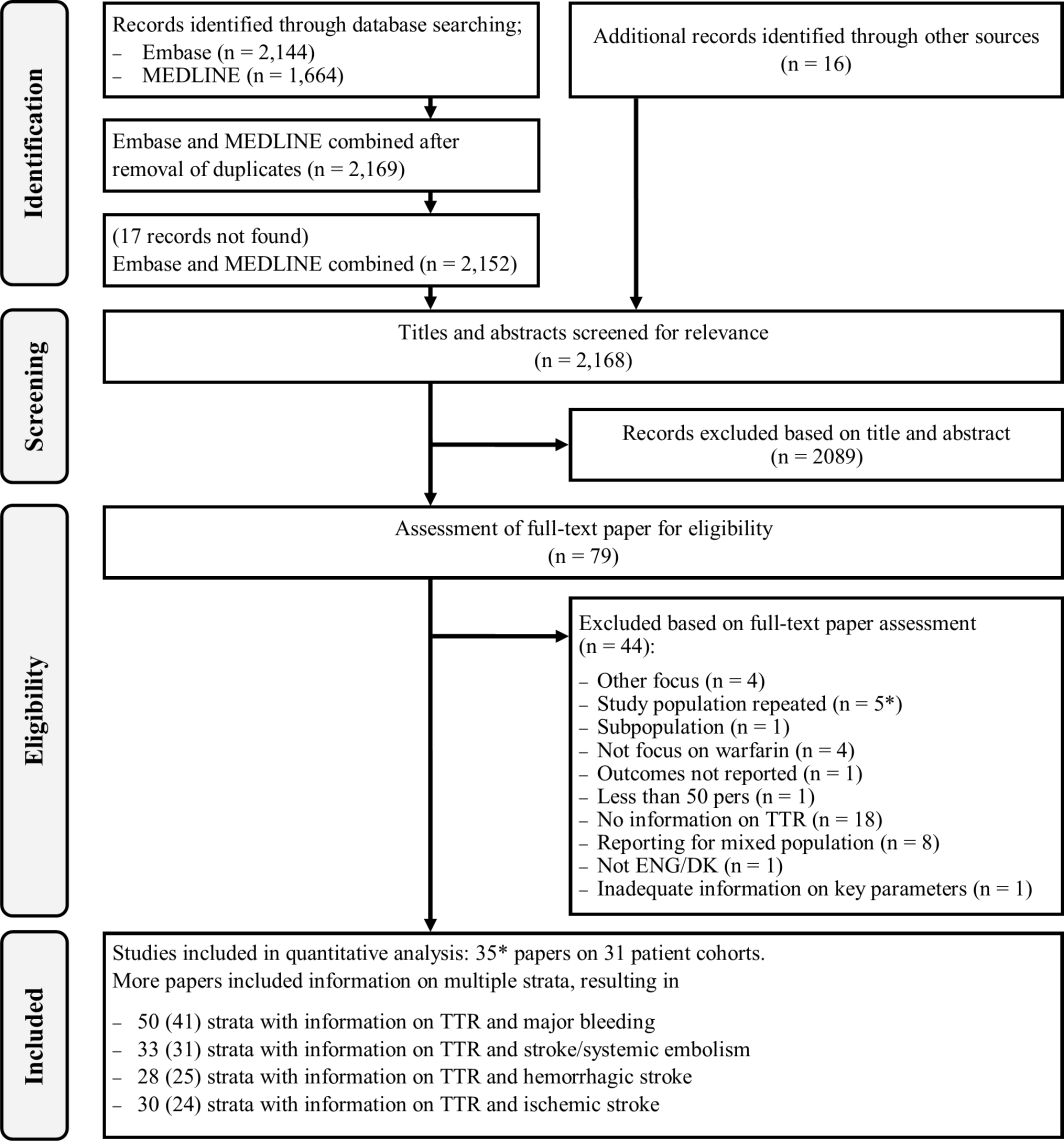
Flowchart of literature search and study exclusions. Adapted from Liberati et al. 2009[10]. Numbers in parenthesis in the lowermost box indicate the number of studies included in random-effects multivariable meta-regressions. Four papers[16,18,19,21] on the same patient cohort were retained as they held information on outcomes of interest not reported in the otherwise included papers on that patient cohort[6,15,17,22].

2144 papers were identified via Embase and 1664 papers were found via MEDLINE. 2169 papers remained after exclusion of duplicates. 17 papers were irretrievable and 16 papers were found via other sources. To reduce the risk of missing relevant papers the results of the literature search were compared with those performed in previous studies with similar research questions[5,9]. No discrepancies were found. 2089 papers were excluded based on their titles and abstract, leaving 79 papers for full-text assessment of eligibility. Further 44 papers did not meet the inclusion criteria, leaving 35 papers with relevant information for inclusion based on 31 patient cohorts; for 4 patient cohorts information was retrieved from different papers as these held data on different outcomes of interest[6,15–20]. A flowchart of the study selection and reasons for exclusions is given in Fig 1 [10].

Papers on 13 RCTs and 18 ‘real-life’ studies were included, of which 23 studies were prospective, seven studies were retrospective, and one was a combination. Six patient cohorts were from North America, 12 from Europe/UK, 12 patient cohorts were multinational, and one was from Turkey. In total, the patient cohorts included more than 100,000 patients with AF on warfarin therapy. More papers reported on multiple treatment strata, which, henceforth, are treated as individual studies. The unweighted estimated mean TTR of the studies was 64% [range: 25–90%], the unweighted mean age was 74 years [range: 64–92], an unweighted average 21% [range: 0–58%] of populations had had a prior stroke/TIA, and included a mean of 41% [range: 0–100%] women. Study characteristics are seen in Table 1.

Publication bias was indicated for the 3rd (highest) tertiles of mean TTR for SSE (*p* = 0.07), HS (*p* = 0.10), and IS (*p* = 0.01) by Egger’s regression statistics, though the basis for analysis was small for HS and IS (number of studies <10). Slight asymmetry was observed for the funnel plots for MB in the 1st and 2nd tertiles of mean TTR, but was not confirmed by Egger’s regression statistics (see S1 Fig). Missing information on prior stroke/TIA was deciding for whether studies could be included in the adjusted analyses. There were no statistically significant differences in outcome rates, mean TTR, number of patient-years, or mean age between the studies that were included in adjusted analyses and those that were not.

**Fig 2:**
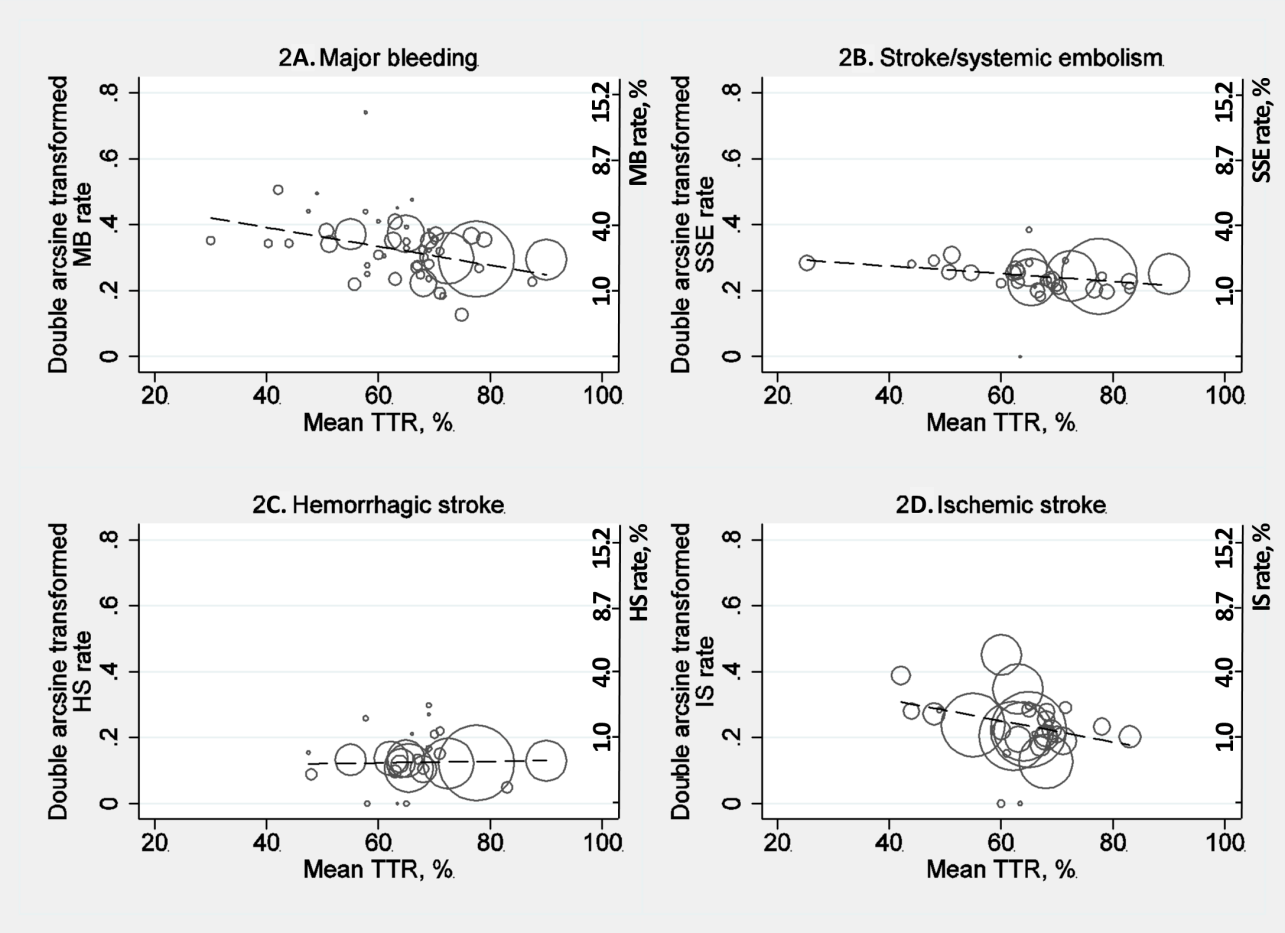
Bubble plots including fitted regression lines. Fitted meta-regression models with mean TTR as predictor of outcomes, respectively hemorrhagic stroke, ischemic stroke, major bleeding, and stroke/systemic embolism. Circles are sized inversely proportionally to the estimated within-study variance of effect reported in studies. The dashed line indicates the fitted regression line. S2 Fig supplies illustration of study weights applied in the random-effects meta-regressions. HS: Hemorrhagic stroke, IS: Ischemic stroke, MB: Major bleeding, SSE: Stroke/systemic embolism, TTR: Time in therapeutic range.

50 studies, i.e. strata, reported on MB rates. In the univariable meta-regression with mean TTR as only predictor for MB, mean TTR was negatively and statistically significantly correlated with the arcsine transformed rate of MB (Fig 2A; *p*<0.01; Table 2). The proportion of residual variance explained by between-study heterogeneity was high (*I*^*2*^_*res*_ = 89.6%)[13]. 41 studies reported on all relevant clinical covariates and were included in the multivariable meta-regression. Mean TTR remained significantly correlated with the transformed MB rate, when adjusting for clinically relevant variables (*p* = 0.03; Table 2) and the proportion of the residual variance caused by between-study heterogeneity was lower (*I*^*2*^_*res*_ = 83.5%). In the univariable meta-regression on the studies included in the adjusted analysis (N = 41), the correlation between mean TTR and the MB rate was in general agreement with the impact observed in the primary univariable meta-regression (Table 2).

**Table 2:** Results of univariable and multivariable random-effects meta-regressions of the impact on outcomes of mean time in therapeutic range.

Outcome*	Meta-regression analysis	Regression basis, N	*I*^*2*^_*res*_	TTR coefficient, 95% CI		
	Univariable	50	89.6%	-0.0029	[-0.0048; -0.0009]	†
**MB**	Univariable‡	41	82.7%	-0.0017	[-0.0034;-0.0000]	†
	Multivariable	41	83.5%	-0.0019	[-0.0036;-0.0002]	†
	Univariable	33	65.9%	-0.0012	[-0.0020;-0.0004]	†
**SSE**	Univariable‡	31	66.5%	-0.0012	[-0.0020;-0.0004]	†
	Multivariable	31	43.2%	-0.0002	[-0.0009;0.0006]	-
	Univariable	28	69.6%	0.0003	[-0.0020;0.0025]	-
**HS**	Univariable‡	25	71.5%	0.0004	[-0.0022;0.0030]	-
	Multivariable	25	69.1%	0.0002	[-0.0017;0.0021]	-
	Univariable	30	94.4%	-0.0032	[-0.0065;0.0000]	-
**IS**	Univariable‡	24	94.4%	-0.0022	[-0.0060;0.0016]	-
	Multivariable	24	92.7%	-0.0015	[-0.0051;0.0021]	-

CI: Confidence interval, HS: Hemorrhagic stroke, IS: Ischemic stroke, MB: Major bleeding, SSE: Stroke/systemic embolism, TTR: Time in therapeutic range

*double arcsine transformed rate of outcome

†*p*<0.05

‡ Univariable meta-regression with mean TTR as only predictor, performed on the subset of studies used in the multivariable meta-regression

33 studies reported on SSE. Increasing mean TTR was significantly associated with a decrease in SSE in the univariable meta-regression (Fig 2B; *p*<0.01; Table 2). Residual variance explained by between-study heterogeneity was considerable (*I*^*2*^_*res*_ = 65.9%). 31 studies composed the basis for the multivariable meta-regression. After adjustment, mean TTR was no longer significantly correlated with SSE (*p* = 0.69; Table 2), but the proportion of residual variance caused by between-study heterogeneity was decreased (*I*^*2*^_*res*_ = 43.2%). The results from the univariable meta-regression on the studies included in the multivariable meta-regression (N = 31) was similar to the primary univariable meta-regression on all studies (Table 2).

### Supplementary analyses

28 studies held information on HS. Upon univariable meta-regression mean TTR was not associated with HS and the proportion of residual variance explained by between-study heterogeneity was considerable (Fig 2C; *p* = 0.81; Table 2; *I*^*2*^_*res*_ = 69.6%). 25 studies on HS held information allowing for multivariable meta-regression, but adjusting for presence of prior stroke/TIA and mean age, did not change the results (*p* = 0.85; Table 2; *I*^*2*^_*res*_ = 60.1%). Results of the univariable meta-regression on the 25 studies were in agreement with the primary univariable meta-regression results (Table 2).

30 studies reported on IS. In the univariable meta-regression, mean TTR was negatively, borderline significantly correlated with the double arcsine transformed IS rate (Fig 2D; *p* = 0.05; Table 2). 24 studies were available for multivariable meta-regression. After adjustment, the correlation remained insignificant (*p* = 0.40; Table 2). The proportion of residual variance caused by between-study heterogeneity was high in both the uni- and multivariable meta-regression (*I*^*2*^_*res*_ = 94.3% and 92.7%, respectively). The results from the univariable meta-regression on studies reporting on all relevant covariates (N = 24) were in general agreement with the results of the primary univariable meta-regression (Table 2).

When adjusting for multiplicity in the multivariable meta-regression on MB, mean TTR remained significantly, negatively correlated with the occurrence of MB[14]. When including relevant clinical and methodological covariates in the meta-regressions, mean TTR was significantly correlated only with the transformed MB rate (*p* = 0.02) and statistical heterogeneity remained for all outcomes (MB *I*^*2*^_*res*_ = 64.7%, SSE *I*^*2*^_*res*_ = 33.8%, HS *I*^*2*^_*res*_ = 67.0%, IS *I*^*2*^_*res*_ = 91.8%; see S4 Table).

**Fig 3:**
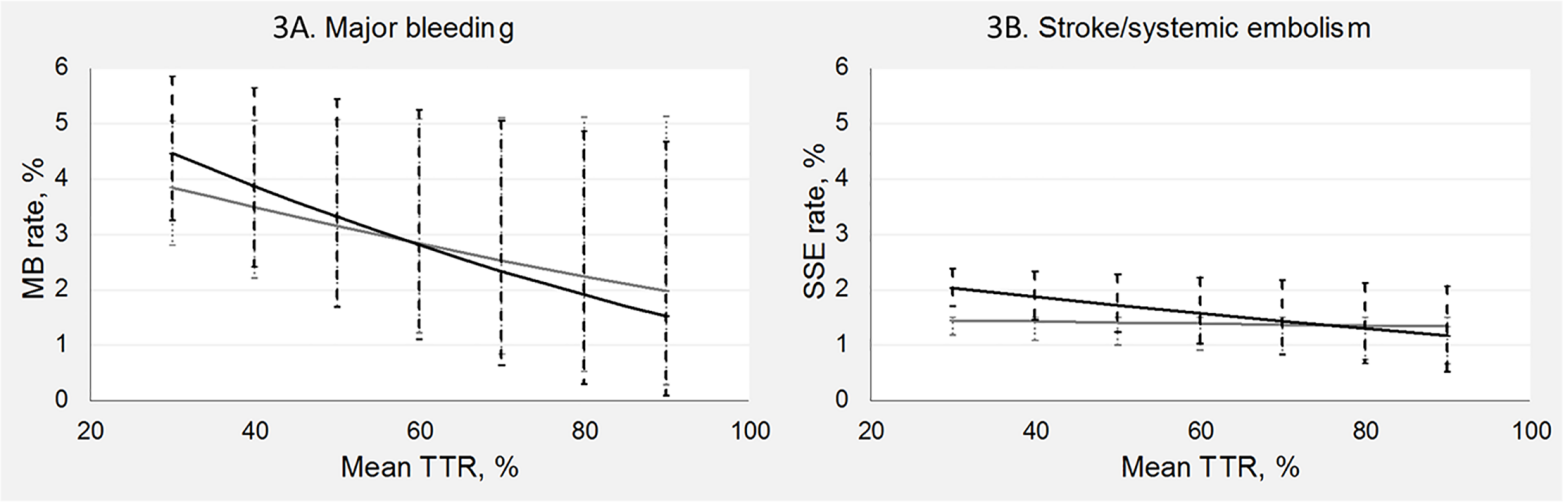
Association between mean time in therapeutic range and rate of major bleeding and stroke/systemic embolism, predicted from uni- and multivariable meta-regressions. Predicted rates of major bleeding and stroke/systemic embolism based on meta-regression with mean TTR as predictor based on univariable (black lines) and multivariable (grey lines) meta-regression. For the predicted rates based on multivariable meta-regression, the unweighted mean values from studies were used for age, proportion with prior stroke/transient ischemic attack, and proportion females. Vertical lines indicate estimated 95% confidence intervals. MB: Major bleeding, SSE: Stroke/systemic embolism, TTR: Time in therapeutic range.

The predicted association between mean TTR and MB and SSE rates from the univariable and multivariable meta-regressions are illustrated in Fig 3A and 3B, respectively. In the adjusted analyses, the predicted correlation between mean TTR and both outcomes is more modest compared to the predicted correlation from the unadjusted analyses.

## Discussion

To our knowledge, this is the first study to evaluate the association between mean TTR and outcomes with adjustment for the clinical characteristics of the patient populations. As expected, increasing mean TTR is associated with a decreasing rate of MB and SSE. The principal findings are that the association is markedly weakened when taking into account differences in relevant clinical characteristics of the included patient cohorts, i.e. mean age, the proportion with prior stroke/TIA, and proportion female of the population, when relevant. Furthermore, the main benefit of increasing mean TTR of a patient population would be reached by lower rates of MB, whereas the effect of increasing mean TTR on the rate of SSE appeared to be limited.

The included papers were published in the period 2005–2015, for which reason patient cohort and treatment characteristics may be assumed to be similar to what may be expected in 2017, thereby likely increasing homogeneity for these parameters and the relevance of the study[50,51].

There appears to be a ‘publication skewness’ with markedly more studies reporting on the safety outcome MB, compared to the effectiveness outcome SSE. This could be an indication of a greater interest in research on safety issues than on effectiveness, though SSE may be equally as devastating as MB. The existence of more papers on MB may also be an explanatory factor, why only analyses found mean TTR to be a significant predictor for this outcome in the adjusted meta-regression model. The availability of fewer studies decreases the robustness of analyses and impedes the establishment of associations[13]. For both IS and HS it could be hypothesized that too few studies were available for meta-regression and the validity of these models is discussable.

The present analyses are based on study level covariates and results are therefore potentially valid only for patient cohorts. Thus, the present analyses do not necessarily reflect the correlation between mean TTR and outcomes for individual patients, but may be used to e.g. estimate the possible benefit of increasing mean TTR for entire patient cohorts. The adjusted models only account for cohorts with characteristics within the value ranges of the used studies. The present results are intended to reflect a broad AF population, including, amongst other, new-starters[23,29] and the very old[39,47], who are known to be at a greater risk of complications than experienced warfarin users and younger patients[8,9]. These subgroups are a part of the AF patient population for whom treatment strategies are decided. Therefore, they should be included in models concerning the association between quality in warfarin therapy for the general AF population and complications. The generalizability of the study results to specific sub-groups is discussable.

According to Sandén et al.[44], the rate of complications during warfarin therapy does not correlate to TTR of centers when the mean TTR is above 70%. Cancino et al.[52] have found the same lack of correlation at high TTRs. As the correlation has been confirmed for data sets with lower TTR levels[7,8,52], this could suggest that the association between mean TTR and complication rates might not be completely linear. In the present model, the double arcsine transformation of the rates was used to normalize the data and stabilize variances. However, in consequence, the predicted association on the original scale is also slightly nonlinear (Fig 3), which agrees with the hypothesis of a nonlinear association.

The present paper suffers from a number of limitations. One author (ASV) performed the literature search, evaluated the papers, and excluded studies based on the pre-specified inclusion criteria. This entails the possibility that some studies have been missed and that studies have been erroneously excluded. Likewise, the data extraction was performed by a single author. Optimally, these tasks should have been undertaken or validated by more authors[10]. The purpose of this study was to accumulate relevant information and evaluate the association between mean TTR of patient populations on warfarin therapy and outcomes, for which reason no formal quality assessment of studies was performed in relation to the present literature review, nor were studies excluded due to insufficient quality. Study design could be hypothesized to affect outcomes[9], but supplementary analyses including methodological covariates did not affect results substantially (see S4 Table). The inclusion of different study designs may be a contributory factor to the high level of between-study heterogeneity observed in this study. However, focusing on e.g. RCTs to decrease between-study heterogeneity would unsettle the foundation of the meta-regression substantially, for which reason this restriction was opted out of.

The definition of outcomes and reporting differ in studies, which hampers their comparability. To allow for meta-regression, the definition of outcomes was used as applied in studies and assumptions were imposed on, for instance, data equivalency and transferability. This likely decreases the validity of the results, and may be an explanatory factor for the high degree of uncertainty pertaining to the results and the large proportion of statistical heterogeneity composed by residual between-study heterogeneity.

A pragmatic approach was taken to the inclusion of covariates in the adjusted meta-regressions, balancing data availability with the request to include all relevant information. Only including a subset of relevant clinical covariates in the meta-regressions most likely subjects the results to unobserved confounding. However, inclusion of more clinically relevant variables would cause the exclusion of further studies in the adjusted analyses, rendering the basis for the present analysis unsound.

Despite the substantial degree of between-study heterogeneity, population characteristics were relatively similar across the included studies, which, in fact, complicates the establishment of correlations between the covariates and outcomes. If a greater dispersion of values for covariates were observed, associations might have been easier to establish. With the present dataset, the resulting coefficients and associated uncertainty may be susceptible to the inclusion of new studies if their data are dissimilar to the data used in the present analysis. The results presented here are based on study level covariates and as such may be subject to ecological bias. The use of aggregate data should incite caution when interpreting the results.[14] However, the association between the covariates used in the adjusted analyses and outcomes has previously been established and the tendencies here are in general agreement with prior findings[2,8]. The uncertainty introduced by ecological fallacy in the present study might be clarified in future analysis, if patient-level data are available[14].

In 2008, Wan et al.[5] performed a systematic review and linear regression analysis on the relationship between TTR and MB and thromboembolism. In agreement with the results by Wan et al., our study indicated a significant inverse correlation between TTR and MB, even when adjusting for differences in patient cohort characteristics. Wan et al.[5] also found a significant inverse relationship between TTR and thromboembolisms. As the present study applied an associated outcome, SSE, the same inverse relationship was expected and confirmed in the univariable meta-regression, but could not be found when adjusting for patient cohort characteristics. The present study includes more studies and takes a different analytical approach to the interpretation of the data by application of the random-effects meta-regression model, which may explain the differences in results.

Cancino et al.[52] have shown differences in the predictive ability of center-level and individual-level mean TTR for complications with individual-level mean TTR being the superior predictor. Individual-level mean TTR may be more appropriate for prediction of complication rates, but center-level mean TTR may still be of use for evaluation of quality improvements[52]. In the present study, no distinction was made between mean TTR summarized at patient- or center-level. Future research based on individual patient-level data may allow for prediction of the impact of improving quality in warfarin therapy with adjustment for differences in patient characteristics and clarify any potential similarities to the models presented here.

Other indicators of quality in warfarin therapy exist that have been linked to the clinical outcomes of patients in warfarin therapy, including the proportion of INR measurements that are within the therapeutic range[53], information on the variability in INR measurements[54], and patterns of anticoagulation control[7]. These indicators of quality in warfarin therapy may also warrant further investigation, as they may provide the basis for improved prediction models for complications during therapy. However, currently, none of these measures have achieved the same widespread use as an indicator of quality in warfarin therapy as the TTR.

In conclusion, mean TTR was inversely and significantly associated with both the occurrence of MB and SSE in the univariable random-effects meta-regression. However, the strength of the association was markedly decreased when adjusting for differences in relevant clinical cohort characteristics. After adjustment, mean TTR remained significantly correlated only with the rate of MB. The present study suggests that increasing quality in warfarin therapy, i.e. increasing mean TTR for patient groups with AF, mainly affects the safety of therapy by decreasing the rate of MB, whereas the rate of SSE does not seem to be substantially reduced with increasing mean TTR. Clinical cohort characteristics should be taken into consideration when evaluating the impact of mean TTR in warfarin therapy on complications. Excluding these from analyses may bias the expected association between mean TTR and outcomes and, for instance, inflate the benefits of increasing quality in warfarin therapy by increasing mean TTR for a patient population, if an ‘optimistic’ correlation is expected.

## Supporting information

S1 Fig Funnel plots and Egger’s regression statistics. (PDF)

S2 Fig Visualization of study weights in random effects meta-regression with mean TTR as predictor of outcomes. (PDF)

S1 Table PRISMA 2009 Checklist. (PDF)

S2 Table Literature search performed in Medline and EMBASE via embase.com. (PDF)

S3 Table Eligibility criteria and rationale. (PDF)

S4 Table Multivariable meta-regression on outcomes with methodological and relevant clinical predictor variables. (PDF)
