# Supplementary material for: The importance of mean time in therapeutic range for complication rates in warfarin therapy of patients with atrial fibrillation: A systematic review and meta-regression analysis
Source: PLoS One. 2017 Nov 20;12(11):e0188482. doi: 10.1371/journal.pone.0188482 (PMC5695846; doi:10.1371/journal.pone.0188482)
Supplement: S1 Fig — (PDF) [file pone.0188482.s001.pdf]

S1 Fig. Funnel plots

Funnel plots with pseudo 95% confidence limits of effect size, i.e. double arcsine transformed rates (of MB, SSE, HS, and IS, respectively), against their estimated standard error for tertiles of mean TTR. Each point indicates a study effect estimate against the estimated standard error. Vertical lines represent the pooled effect within the mean TTR tertile.

There was an indication of small-study effect for the third (highest) tertiles of mean TTR for the outcomes SSE (p=0.07), HS (p=0.10), and IS (p=0.01), respectively, see Table S1 Fig. The basis for analysis for the outcomes HS and IS was small (number of studies within mean TTR tertile <10). Furthermore, slight, non-significant symmetry was observed for MB in the first and second mean TTR tertile.

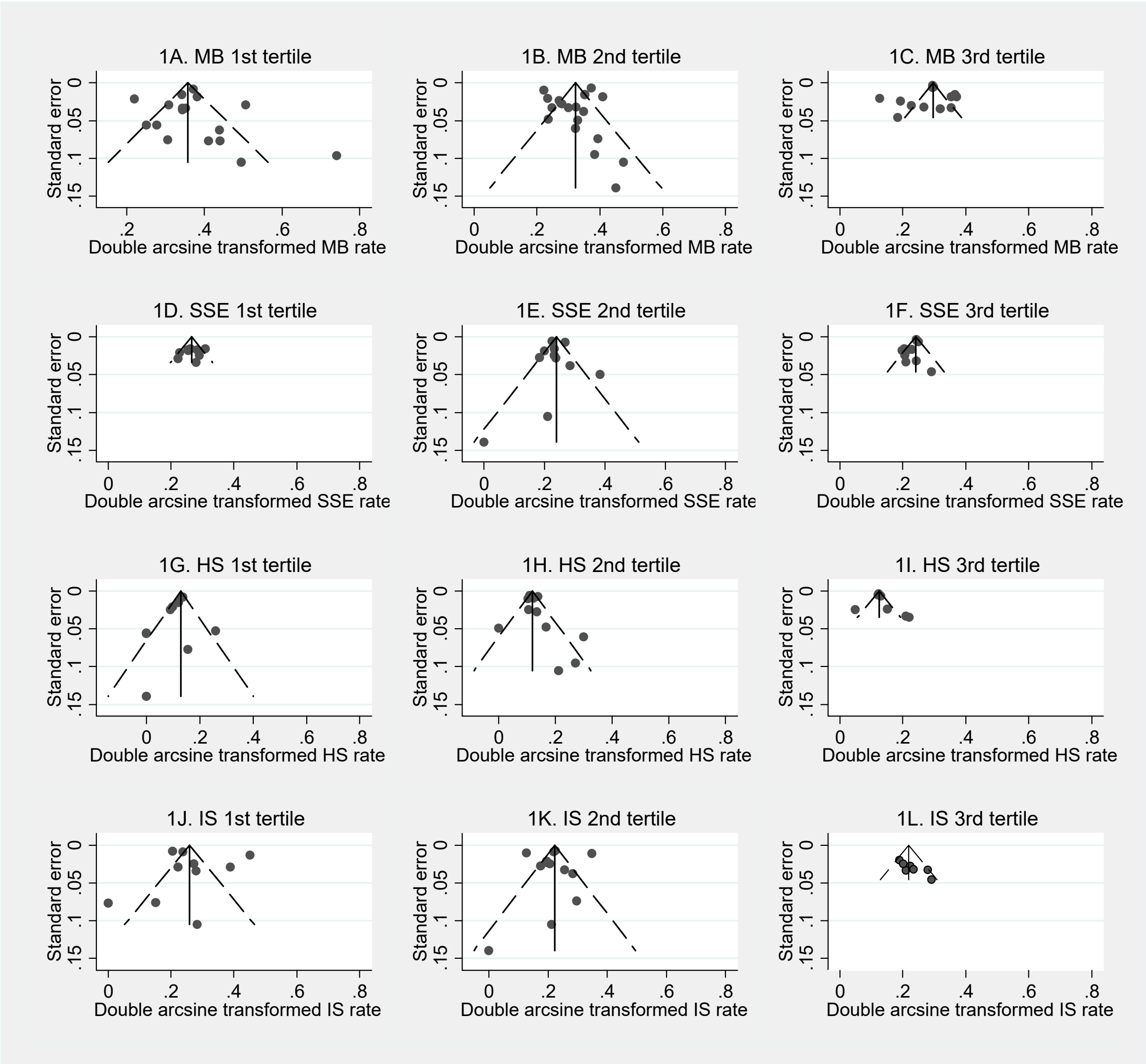

HS: Hemorrhagic stroke, IS: Ischemic stroke, MB: Major bleeding, SSE: Stroke/systemic embolism

Table S1 Fig. Information on mean TTR tertiles

	**Tertile**		
**MB**	**1st**	**2nd**	**3rd**
Studies, N	17	20	13
Range of mean TTR within tertile	[30.0%-61.0%]	[62.6%-69.0%]	[70.0%-90.0%]
Average mean TTR within tertile	51.6%	66.4%	76.1%
Egger's regression statistics	p=0.78	p=0.68	p=0.46
**SSE**	**1st**	**2nd**	**3rd**
Studies, N	11	11	11
Range of mean TTR within tertile	[25.3%-63.0%]	[63.4%-69.0%]	[70.0%-90.0%]
Average mean TTR within tertile	53.1%	66.3%	77.4%
Egger's regression statistics	p=0.52	p=0.84	p=0.07†
**HS**	**1st**	**2nd**	**3rd**
Studies, N	10	11	7*
Range of mean TTR within tertile	[47.5%-63.8%]	[64.0%-69.0%]	[70.0%-90.0%]
Average mean TTR within tertile	57.7%	66.8%	76.4%
Egger's regression statistics	p=0.19	p=0.34	p=0.10†
**IS**	**1st**	**2nd**	**3rd**
Studies, N	10	13	7*
Range of mean TTR within tertile	[42.1%-62.2%]	[63.0%-68.0%]	[68.1%-83.0%]
Average mean TTR within tertile	54.1%	65.6%	72.9%
Egger's regression statistics	p=0.70	p=0.94	p=0.01†

HS: Hemorrhagic stroke, IS: Ischemic stroke, MB: Major bleeding, SSE: Stroke/systemic embolism, TTR: Time in therapeutic range, percentage

*Basis for funnel plots and Egger's regression statistics < 10 studies

†Statistical significance for p<0.10 used for Egger's regression statistics
