# Supplementary material for: The importance of mean time in therapeutic range for complication rates in warfarin therapy of patients with atrial fibrillation: A systematic review and meta-regression analysis
Source: PLoS One. 2017 Nov 20;12(11):e0188482. doi: 10.1371/journal.pone.0188482 (PMC5695846; doi:10.1371/journal.pone.0188482)
Supplement: S2 Fig — (PDF) [file pone.0188482.s002.pdf]

S2 Fig. Visualization of study weights in random effects meta-regression with mean TTR as predictor of outcomes

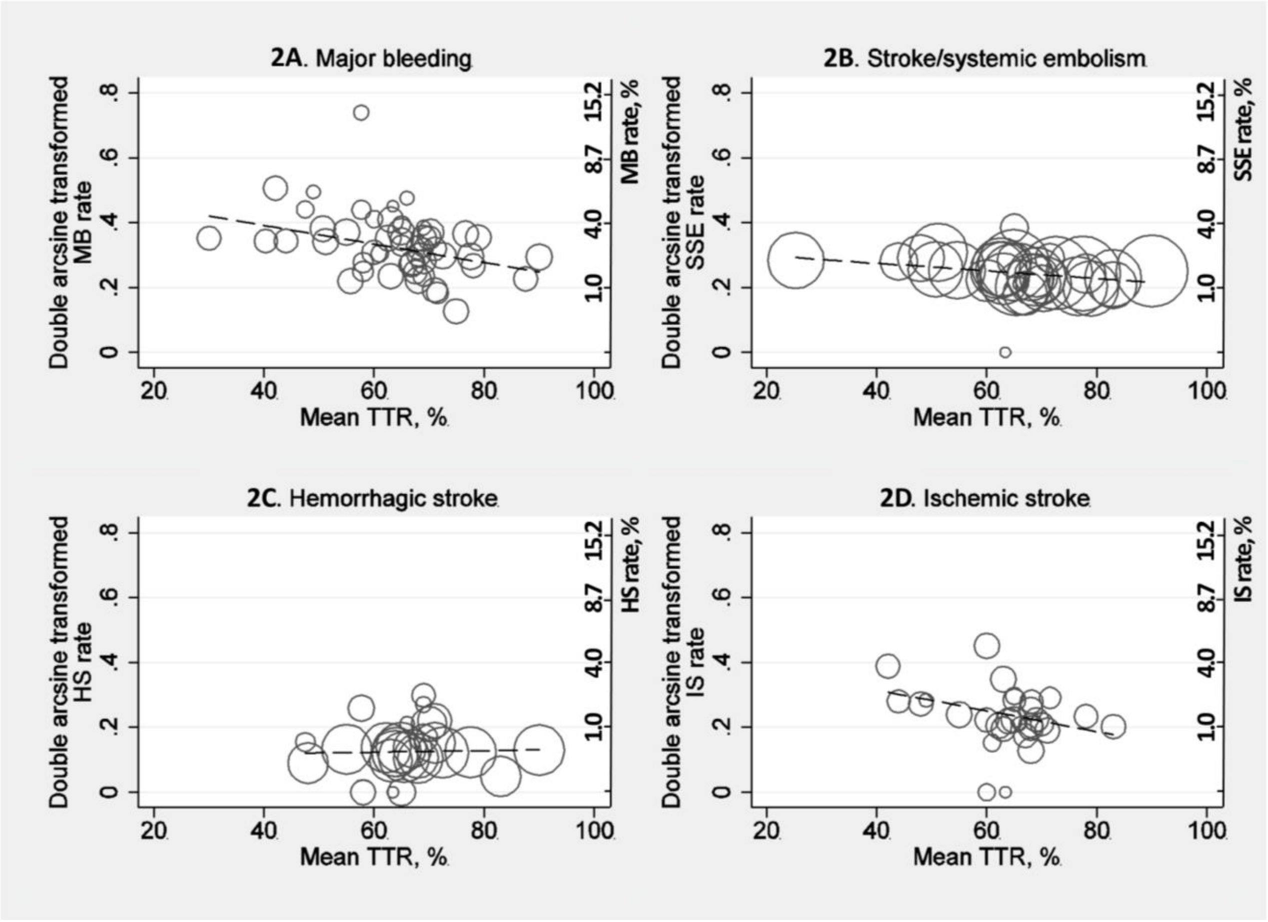

The size of circles represents total variance, i.e. within- and between-study variance, and is proportional to the weight of the studies in analyses. The dashed line indicates the fitted regression model. HS: Hemorrhagic stroke, IS: Ischemic stroke, MB: Major bleeding, SSE: Stroke/systemic embolism, TTR: Time in therapeutic range.
