# Supplementary material for: The importance of mean time in therapeutic range for complication rates in warfarin therapy of patients with atrial fibrillation: A systematic review and meta-regression analysis
Source: PLoS One. 2017 Nov 20;12(11):e0188482. doi: 10.1371/journal.pone.0188482 (PMC5695846; doi:10.1371/journal.pone.0188482)
Supplement: S2 Table — (PDF) [file pone.0188482.s004.pdf]

S2 Table. Literature search performed in Medline and EMBASE via embase.com

<table>
<tr><th>Search engine</th><th>Date of query</th></tr>
<tr><td>Medline</td><td>2 February 2016</td></tr>
<tr><td colspan="2">'atrial fibrillation'/exp OR 'atrial fibrillation' AND ('anticoagulant agent'/exp OR 'anticoagulant agent' OR anticoagulants OR 'vitamin k antagonist' OR 'international normalized ratio'/exp OR 'international normalized ratio') AND ('warfarin'/exp OR 'warfarin' OR warfarin) AND ('clinical article'/exp OR 'clinical article' OR 'clinical trial'/exp OR 'clinical trial' OR 'cohort analysis'/exp OR 'cohort analysis' OR 'observational study'/exp OR 'observational study' OR 'retrospective studies'/exp OR 'retrospective studies' OR 'prospective study'/exp OR 'prospective study' OR 'major clinical study'/exp OR 'major clinical study' OR 'intervention study'/exp OR 'intervention study') AND ('thrombosis'/exp OR 'thrombosis' OR thrombosis OR 'bleeding'/exp OR 'bleeding' OR bleeding OR hemorrhage) AND [2005-2015]/py AND ([danish]/lim OR [english]/lim) AND [humans]/lim AND ('article'/it OR 'article in press'/it) AND [medline]/lim</td></tr>
<tr><td>Embase</td><td>2 February 2016</td></tr>
<tr><td colspan="2">'atrial fibrillation'/exp OR 'atrial fibrillation' AND ('anticoagulant agent'/exp OR 'anticoagulant agent' OR anticoagulants OR 'vitamin k antagonist' OR 'international normalized ratio'/exp OR 'international normalized ratio') AND ('warfarin'/exp OR 'warfarin' OR warfarin) AND ('clinical article'/exp OR 'clinical article' OR 'clinical trial'/exp OR 'clinical trial' OR 'cohort analysis'/exp OR 'cohort analysis' OR 'observational study'/exp OR 'observational study' OR 'retrospective studies'/exp OR 'retrospective studies' OR 'prospective study'/exp OR 'prospective study' OR 'major clinical study'/exp OR 'major clinical study' OR 'intervention study'/exp OR 'intervention study') AND ('thrombosis'/exp OR 'thrombosis' OR thrombosis OR 'bleeding'/exp OR 'bleeding' OR bleeding OR hemorrhage) AND [2005-2015]/py AND ([danish]/lim OR [english]/lim) AND [humans]/lim AND ('article'/it OR 'article in press'/it) AND [embase]/lim</td></tr>
</table>
