# Supplementary material for: The importance of mean time in therapeutic range for complication rates in warfarin therapy of patients with atrial fibrillation: A systematic review and meta-regression analysis
Source: PLoS One. 2017 Nov 20;12(11):e0188482. doi: 10.1371/journal.pone.0188482 (PMC5695846; doi:10.1371/journal.pone.0188482)
Supplement: S3 Table — (PDF) [file pone.0188482.s005.pdf]

**S3 Table. Eligibility criteria and rationale**

The present eligibility criteria are similar to criteria applied in previous systematic reviews and meta-analyses[1,2], increasing the comparability of results.

| Criterion | Rationale |
|---|---|
| **Population** | |
| Main indication for anticoagulant therapy: Non-valvular atrial fibrillation | Anticoagulant therapy may be provided in response to multiple indications that entail different prognoses. However, the focus of the present study is on non-valvular AF. Hence, the main indication should be non-valvular AF though patients included in studies may also have other indications for anticoagulant therapy.<br><br>The treatment recommendations, target INR range, and risk profile for valvular AF is different from that for non-valvular AF, necessitating the restricted focus on *non*-valvular AF to increase homogeneity of the population. |
| Adult patients, age>18 years | To increase homogeneity of the population and to best represent the patient population observed in practice. |
| *Exclusion*:<br>Mixed populations | Studies reporting aggregated results for patients with mixed indications, e.g. AF and DVT, as they have different prognoses and characteristics. |
| *Exclusion*:<br>subpopulations | The purpose was to identify the correlation between mean TTR and complications in the broader AF patient population. Studies on subgroups, e.g. patients with diabetes, renal impairment, or cancer were excluded as the results of such studies were not expected to be transferable to the general AF patient population. |
| **Intervention** | |
| Mainly used vitamin K antagonist: warfarin | Anticoagulant therapy may be provided through the use of more types of VKAs that demonstrate different pharmacological properties, e.g. different half-lives and drug interaction profiles, which consequently may affect their safety, effectiveness, and management.<br><br>Some studies report on a mix of VKAs. To increase comparability and homogeneity across the extracted data, the most commonly used VKA in studies should be warfarin. Studies in which only a minority of patients were treated with other VKAs were included. |
| Only dose-adjusted warfarin therapy | To maintain safety and effectiveness of warfarin, medication dose-adjustment should be performed to reflect patients' need of anticoagulant therapy. Dose-adjustments are recommended[3] and is the more commonly used practice, compared to fixed-dose warfarin therapy. |
| Target therapeutic range; international normalized ratio of 2-3 | The generally recommended target therapeutic INR range for warfarin therapy for patients with AF is 2-3[3]. For patients with another target range, another risk profile would be observed with respect to the risk |

| | |
|---|---|
| | of hemorrhage and thromboembolism[4], making data on safety and effectiveness at different TTR levels incomparable. |
| **<u>C</u>omparators** | The ‘C’ of the PICOS search strategy is omitted as the comparator is irrelevant. The purpose of the present study was to retrieve studies with information on outcome rates for patients in warfarin therapy, for which the TTR is stated, irrespective of the comparator. |
| Information on TTR required | The purpose of the present study was to investigate the correlation between the quality of warfarin therapy, i.e. mean TTR of patient populations, and outcome rates, for which reason information on the TTR should be stated. |
| *Exclusion*: Anticoagulation in relation to other therapies or surgery | Studies were excluded if the safety/effectiveness of warfarin therapy were investigated in relation to other therapies or surgery, for which situations the risk of adverse hemorrhagic or thromboembolic events would be affected. Such situations would not be representative of the risk profile of the general AF patient population in ‘normal’ warfarin therapy, which was the main focus of the present study. |
| **<u>O</u>utcomes** | Studies that did not report on any type of either hemorrhage or thromboembolism were excluded as they would not make for the quantitative analyses of the present study. The outcome measures should be quantified. |
| Hemorrhage | Outcome for the present study. Excess anticoagulant therapy increases the risk of hemorrhages, which thus may occur as a consequence of suboptimal anticoagulant therapy. |
| Thromboembolism | Outcome for the present study. Insufficient anticoagulant therapy leaves the patient at an increased risk of thromboembolism, which thus may occur as a consequence of suboptimal anticoagulant therapy. |
| **<u>S</u>tudy characteristics** | |
| Prospective/retrospective | Both prospective and retrospective studies were considered to potentially provide data for the present analysis and were therefore considered eligible. |
| RCTs and observational cohort studies* | Both RCTs and observational cohort studies were considered to potentially provide data for the present analysis and were therefore considered eligible. Studies should represent original research. When more studies reported on the same patient population, only data from one study were used in analyses to avoid inclusion of the same study population more than once for each analysis.<br><br>In general, outcome rates would be expected to be lower in RCTs compared to those in observational cohort studies[2], but this might be mediated through a higher quality in warfarin therapy under the controlled design, which might be reflected in the TTR. Likewise, anticoagulation clinics may deliver higher-quality therapy than community practice[2], but this may also be mediated through a higher TTR. |

| | |
|---|---|
| | *Reviews and meta-analyses were used in the execution of the present study, but were not included in the systematic review, which constituted the data basis for the quantitative analyses. |
| >50 individuals included in each treatment group | Though increased compared to that of a similar population not in anticoagulant therapy, the rate of thromboembolism and hemorrhage is relatively low in the AF population treated with warfarin therapy. To increase the validity of possible zero-findings in the included studies, at least 50 individuals should be included in each treatment group. |
| Mean follow-up of patients > 3 months | Though increased compared to that of a similar population not in anticoagulant therapy, the rate of thromboembolism and hemorrhage is relatively low in the AF population treated with warfarin therapy. To increase the validity of possible zero-findings in the included studies, the mean follow-up of patients in studies should be at a minimum of 3 months. |
| Published<br>Jan 2005 – Dec 2015 | Restricted to include studies published within approximately the last decade to ensure comparability in treatment regimens to what would expectedly be observed today and to avoid Will Rogers phenomenon[5,6]. |
| Full-text | All information deemed relevant for publication should be available. |
| Danish/English | To ensure complete understanding of the written content. We did not have the resources to translate studies written in other languages. |

**References**


[1] Wan Y, Heneghan C, Perera R, Roberts N, Hollowell J, Glasziou P, et al. Anticoagulation control and prediction of adverse events in patients with atrial fibrillation: a systematic review. Circ Cardiovasc Qual Outcomes 2008;1:84–91.

[2] Mearns ES, White C, Kohn CG, Hawthorne J, Song J-S, Meng J, et al. Quality of vitamin K antagonist control and outcomes in atrial fibrillation patients: a meta-analysis and meta-regression. Thromb J 2014;12:14.

[3] Kirchhof P, Benussi S, Kotecha D, Ahlsson A, Atar D, Casadei B, et al. 2016 ESC Guidelines for the management of atrial fibrillation developed in collaboration with EACTS. Eur Heart J 2016;37:2893–962.

[4] Amouyel P, Mismetti P, Langkilde LK, Jasso-Mosqueda G, Nelander K, Lamarque H. INR variability in atrial fibrillation: A risk model for cerebrovascular events. Eur J Intern Med 2009;20:63–9.

[5] Delgado-Rodríguez M, Llorca J. Bias. J Epidemiol Community Health 2004;58:635–41.

[6] Eckman MH, Singer DE, Rosand J, Greenberg SM. Moving the tipping point the decision to anticoagulate patients with atrial fibrillation. Circ Cardiovasc Qual Outcomes 2011;4:14–21.
