# Supplementary material for: The importance of mean time in therapeutic range for complication rates in warfarin therapy of patients with atrial fibrillation: A systematic review and meta-regression analysis
Source: PLoS One. 2017 Nov 20;12(11):e0188482. doi: 10.1371/journal.pone.0188482 (PMC5695846; doi:10.1371/journal.pone.0188482)
Supplement: S4 Table — (PDF) [file pone.0188482.s006.pdf]

S4 Table. Multivariable meta-regression on outcomes with methodological and relevant clinical predictor variables

Outcome*	Regression basis, N	I^2_{res}	Time in therapeutic range, coefficient, 95% CI		
**Major bleeding**	41	64.7%	-0.0020	[-0.0036;-0.0004]	†
**Stroke/systemic embolism**	31	33.8%	-0.0006	[-0.0018;0.0006]	
**Hemorrhagic stroke**	25	67.0%	0.0014	[-0.0020;0.0048]	
**Ischemic stroke**	24	91.8%	-0.0022	[-0.0057;0.0012]	

Impact of mean TTR on double arcsine transformed outcome rates estimated from multivariable meta-regression with methodological and relevant clinical predictor variables. The variable for proportion of patient population that is female sex was omitted for major bleeding and hemorrhagic stroke, as it is not a known risk factor for bleeding. Publication years 2005 to 2007, setting; anticoagulation clinic, prospective design and multinational location used as baseline. When relevant, variables were omitted in analyses due to collinearity. CI: Confidence interval.

*double arcsine transformed outcome rates

†$p<0.05$
